# Study of the Myosin Relay Helix Peptide by Molecular Dynamics Simulations, Pump-Probe and 2D Infrared Spectroscopy

**DOI:** 10.3390/ijms25126406

**Published:** 2024-06-10

**Authors:** Holly Freedman, Jack A. Tuszynski

**Affiliations:** 1 Center for Molecular Spectroscopy and Dynamics, Institute for Basic Science (IBS), Seoul 02841, Republic of Korea; 2 Department of Chemistry, Korea University, Seoul 02841, Republic of Korea; 3 Department of Medicinal Chemistry, College of Pharmacy, University of Utah, 2000 East 30 South Skaggs 306, Salt Lake City, UT 84112, USA; 4 Department of Physics, University of Alberta, 11335 Saskatchewan Dr NW, Edmonton, AB T6G 2M9, Canada; jackt@ualberta.ca; 5 DIMEAS, Politecnico di Torino, Corso Duca degli Abruzzi 24, I-1029 Turin, Italy; 6 Department of Data Science and Engineering, The Silesian University of Technology, 44-100 Gliwice, Poland

**Keywords:** Davydov soliton, myosin, 2D infrared spectroscopy, pump-probe infrared spectroscopy, alpha-helix, ATP hydrolysis, protein energy transfer, molecular dynamics simulations

## Abstract

The Davydov model was conjectured to describe how an amide I excitation created during ATP hydrolysis in myosin might be significant in providing energy to drive myosin’s chemomechanical cycle. The free energy surfaces of the myosin relay helix peptide dissolved in 2,2,2-trifluoroethanol (TFE), determined by metadynamics simulations, demonstrate local minima differing in free energy by only ~2 kT, corresponding to broken and stabilized hydrogen bonds, respectively. Experimental pump-probe and 2D infrared spectroscopy were performed on the peptide dissolved in TFE. The relative heights of two peaks seen in the pump-probe data and the corresponding relative volumes of diagonal peaks seen in the 2D-IR spectra at time delays between 0.5 ps and 1 ps differ noticeably from what is seen at earlier or later time delays or in the linear spectrum, indicating that a vibrational excitation may influence the conformational state of this helix. Thus, it is possible that the presence of an amide I excitation may be a direct factor in the conformational state taken on by the myosin relay helix following ATP hydrolysis in myosin.

## 1. Introduction

Conformational changes in helical structure instigated when a nucleotide triphosphate (NTP) molecule bound to a protein is hydrolyzed can be instrumental in allowing the protein to extract energy from the NTP hydrolysis reaction in order to perform a cellular function [1]. The interchange between two forms of the relay helix belonging to the motor domain of myosin is one example of a protein conformational change that can be influenced by the phosphorylation state of the bound nucleotide. Myosin has a globular head region containing the motor domain, which takes sequential steps along an actin filament, and an extended coiled-coil tail region that binds to the cargo-binding domain or cellular target [2,3]. A converter region connects these two regions and forms part of the lever arm, which amplifies small conformational changes in the motor domain by rotating to produce the power stroke responsible for muscle contraction [4].

A bent to straight transition of the relay helix is at the core of the ATP hydrolysis-driven cycle of binding and unbinding of myosin to actin, and the associated conversion of chemical energy into mechanical work via the power stroke. This transition is crucial to the rotation angle of the lever arm and is linked to the closing of the cleft in the actin-binding region that allows myosin to bind strongly to actin [4,5]. When ATP is bound to the nucleotide-binding site, interactions of the nucleotide with a protein motif known as switch I open the actin-binding cleft [6], causing a kink in the relay helix (M** state), an upward position of the converter region and lever arm, and weakening of the binding of myosin to actin [5]. On the other hand, when ADP occupies the nucleotide-binding site, the actin-binding cleft is closed, the relay helix has a predominantly straight conformation (M* state), the converter region and lever arm have a downward position, and myosin binds tightly to actin [4].

A productive step of myosin requires that the protein remain bound to actin during the power stroke. Thus, while the phosphorylation state of the bound nucleotide can influence the conformation of the relay helix in myosin, processivity requires that a conformation that binds to the cytoskeletal filament exist both before and after the ADP-bound protein is formed. Indeed, experimental studies demonstrate that the phosphorylation state of the nucleotide does not strictly determine the conformation of the relay helix [7], and rather, in the ATP and ADP-P_i_ states for which the relay helix is mainly bent, the relay helix is in a dynamic mixture of straight and bent states [8,9]. Moreover, in its ATPase cycle, myosin is believed to bind to actin while in the ADP-P_i_ bound state [4,5,10], despite the fact that this state favors the bent conformation of the relay helix and weak binding to actin [8]. It is possible that the vibrational energy released during hydrolysis influences the configuration of the relay helix by straightening it, and in this way ensures that myosin remains bound to actin.

It is not known how the energy released during nucleotide hydrolysis—about 12 kcal/mol (4200 cm^−1^ [11])—contributes to protein motor function or other cellular work [12], although this issue is crucial to understanding energy utilization by the human body, in which ATP hydrolysis is the most frequently occurring reaction. This energy is close in magnitude to the energy of two amide I vibrations (3300 cm^−1^). Energy conversion to mechanical work occurs with about 50% efficiency [12]. During ATP hydrolysis, specific molecular vibrations are created. However, within a short time, these vibrations decay into a thermal mixture of lower energy vibrations (a heat bath). Once thermalized, the efficiency of the released energy becomes small and its possible impact on protein or orthophosphate dynamics diminishes since the second law of thermodynamics places a fundamental limit on the conversion of heat to work [13]. That is, any efficient work done by the vibrations created during ATP hydrolysis must be achieved prior to thermalization. It was suggested by Davydov that two amide I modes are created and are involved in an energy transfer process leading to protein conformational changes that facilitate myosin’s chemomechanical cycle [14]. In the original theory of Davydov, a vibrational excitation was proposed to be self-trapped via interaction between the quantum localization of the vibrational excitation and mechanical deformations of an α-helix, thus prolonging its lifetime, and it was proposed to move sequentially in soliton form along the peptide main chain [15,16]. Long-lived vibrational self-trapping of this form has been ruled out at biologically relevant temperatures [17,18,19]. However, it is still possible that a vibrational excitation may move between amide I sites in a protein for a short time span of up to a few picoseconds [20], and that, during this time, it may impact helical structure [21,22,23,24,25,26]. 

**Figure 1:**
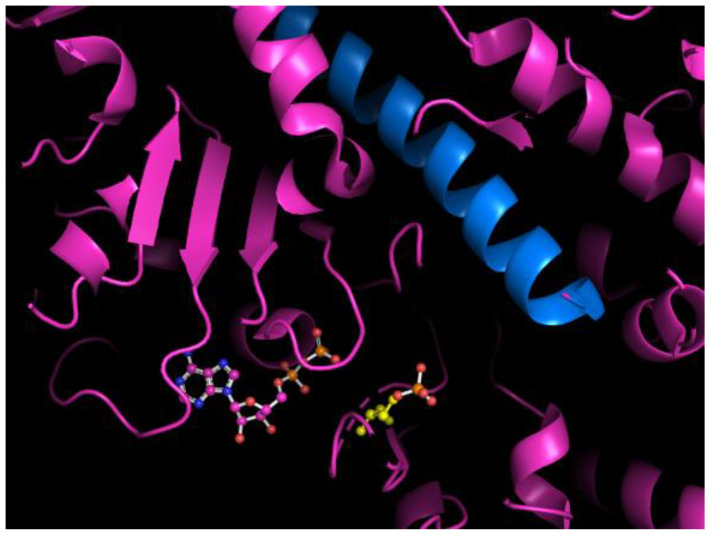
Cartoon representation showing the proximity of the myosin active site bound by an ADP ligand displayed in ball-and-stick mode and of an orthophosphate molecule bound at the entrance to the exit tunnel near residue S217 (myosin V numbering), which is also in ball-and-stick mode and in red, to the relay helix in blue (PDB ID 4PJJ [2]). Residue S217 at the entrance to the exit tunnel is shown in yellow and in ball-and-stick mode. The myosin relay helix is oriented with its N-terminus on the right-hand side of the figure. Rendering was performed using Pymol [29].

Another previous conjecture is that the energy released during ATP hydrolysis may facilitate the translation of the orthophosphate reaction product out of the active site during the hydrolysis-driven cycle [10]. Alternatively, the energy released might influence the dynamics of both the orthophosphate molecule and the protein. Regarding this possibility, a phosphate release state of myosin has been identified by crystallography in which orthophosphate has approached the relay helix to within 6 Å, just before the exit tunnel [2] (see Figure 1). *Ab initio* molecular dynamics simulations have indicated that, during ATP hydrolysis in actin, a new hydroxyl bond formed in the orthophosphate molecule during hydrolysis may be vibrationally excited [27], and, if this happens in myosin, then the phosphate release state might help to facilitate the transfer of vibrational energy from orthophosphate to the relay helix. Indeed, the catalytic mechanism of ATP hydrolysis in myosin is believed to be similar to that in actin; in both proteins, the reaction is dissociative, involves two water molecules in proton transfers, and results in the creation of the double-protonated phosphate H_2_PO_4_^−^ [28].

If there is a direct effect of a vibrational excitation on protein dynamics, what kinds of alterations in protein structure can arise? Does the excitation supply the activation energy for changes in the secondary structure, stabilize the secondary structure, or in other ways impact essential hydrogen bonds or other intramolecular or intermolecular interactions? Computational work [25] has previously investigated whether the interactions between an excited amide I vibration in a protein α-helix and main-chain hydrogen bond lengths could potentially straighten the helix or lead to its increased helicity. This could occur by interactions of the excitation with main-chain hydrogen bonds, causing changes in their lengths and thus the straightening of the helix. In that work, only a very minimal effect on helicity was found [25]. In the past few decades, 2D infrared spectroscopy has arisen as a powerful tool, which can detect picosecond timescale molecular dynamics of biophysical processes [30,31]. We apply this technique to test the effect of a vibrational excitation on the transitions of myosin’s relay helix between bent and straight conformers. To help interpret these results, computational metadynamics simulations are performed to examine the conformational flexibility of this peptide, and FTIR and 2D-IR spectra are computed for the bent and straight conformers. 

## 2. Results

### 2.1. Experimental UV Circular Dichroism and Linear Amide I Spectra

**Figure 2:**
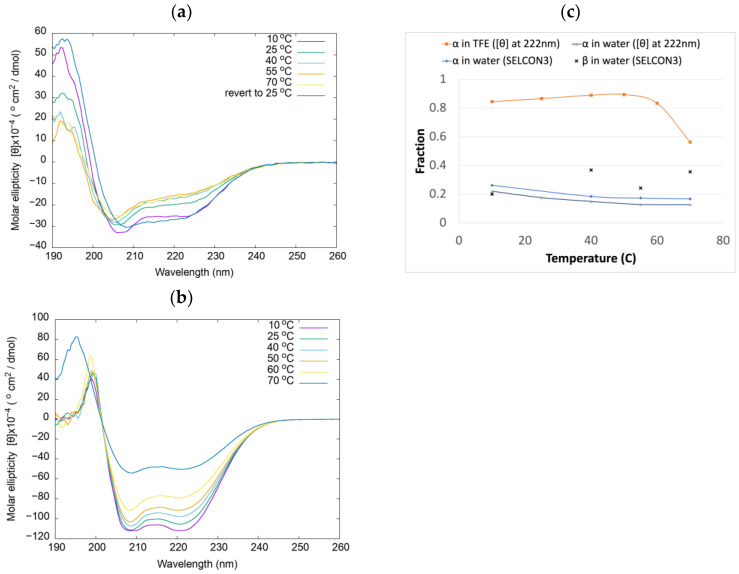
UV circular dichroism (CD) spectra of the myosin relay helix peptide dissolved in (**a**) water, and (**b**) 2,2,2-trifluoroethanol (TFE), shown in units of molar ellipticity [θ]. (**c**) Fractional helicity of the myosin relay helix peptide as a function of temperature in water and TFE, and fractional beta strand structure of the peptide in water.

The temperature-dependent UV circular dichroism (CD) spectra of the myosin relay helix peptide dissolved in water at a concentration of 1.3 g/L are shown in Figure 2a. We analyzed the CD spectra for secondary structure content both with the relationship previously developed by Baldwin and coworkers [32,33] for helical fraction as a function of molar ellipticity per residue at 222 nm ([*θ*]_222_) and with the DichroWeb program [34,35] using the SELCON3 [36,37] (“Set 7” [38,39]) method (Figure 2c). Although the peptide has very little α-helical content in water due to entropic considerations, the α-helical content slightly increases as the temperature is decreased, varying from 0.17 to 0.26 according to SELCON3, indicating that the helical structure is enthalpically favored in water. The results for the peptide in water based on [θ]_222_ closely parallel those from SELCON3. The predicted numbers of helices and helical lengths for the peptide in water vary from 0.8 to 1.1 and from 7.7 to 8.6, respectively, and are plotted in Figure S1. The fractional content of beta strands of the peptide in water predicted by SELCON3 in water varies from 0.2 to 0.4, depending upon the temperature (Figure 2c). When the peptide in water is returned to a temperature of 25 °C from 70 °C, the predicted helical content is 0.28 according to SELCON3, demonstrating that annealing increases the helical content; the peptide is predicted to have 1.1 helices and an average helical length of 8.9 residues. 

To measure the FTIR spectrum of the peptide, a higher concentration of the peptide in solution was needed, and measurements were performed at a concentration of 9 g/L in D_2_O. However, the protein appeared to aggregate in D_2_O, and peaks were seen in the FTIR spectrum characteristic of beta sheets and protein turns. To stabilize the helical structure, the peptide was dissolved in 2-2-2 trifluoroethanol (TFE), which was previously found to stabilize protein secondary structure [40,41,42]. Previous molecular dynamics simulations elucidated the molecular mechanism of the stabilization of peptide α-helices in TFE compared to that in water [41,43,44]. Compared to water, which acts as a hydrogen bond acceptor and donor for peptide main chain atoms, TFE forms weak, non-specific interactions with peptide atoms [43,44]. Thus, extended conformations of the peptide are disfavored since peptide–solvent interactions are less significant. Instead, the lower dielectric constant of TFE compared to water strengthens intramolecular interactions, and the α-helical peptide conformation, which is characterized by intramolecular hydrogen bonding, is favored [41,43,44]. 

The temperature-dependent CD spectra of the peptide in TFE at a concentration of 4 g/L are plotted in Figure 2b up to a temperature of 70 °C (the boiling point of TFE is 78 °C). We plotted fractional helicity based on [θ]_222_ [32,33] for the peptide in TFE, as shown in Figure 2c. The fractional helicity of the peptide in TFE is significantly greater than in water. The predicted fractional content of the peptide is 0.87 helical at 25 °C. Due to the poor quality of our data at lower wavelengths, we were only able to apply DiChroweb to the CD spectrum for the peptide in TFE at the temperature of 70°. The fractional helicity of the peptide in TFE is 0.53 at 70 °C, as predicted by SELCON3, which is in close agreement with the value of 0.56 based on [θ]_222_. At 70 °C, the peptide dissolved in TFE is predicted to contain 2.0 α-helices, the average length of a helix is predicted to be 9.0 residues, and the fractional content of beta strands is 0.05.

**Figure 3:**
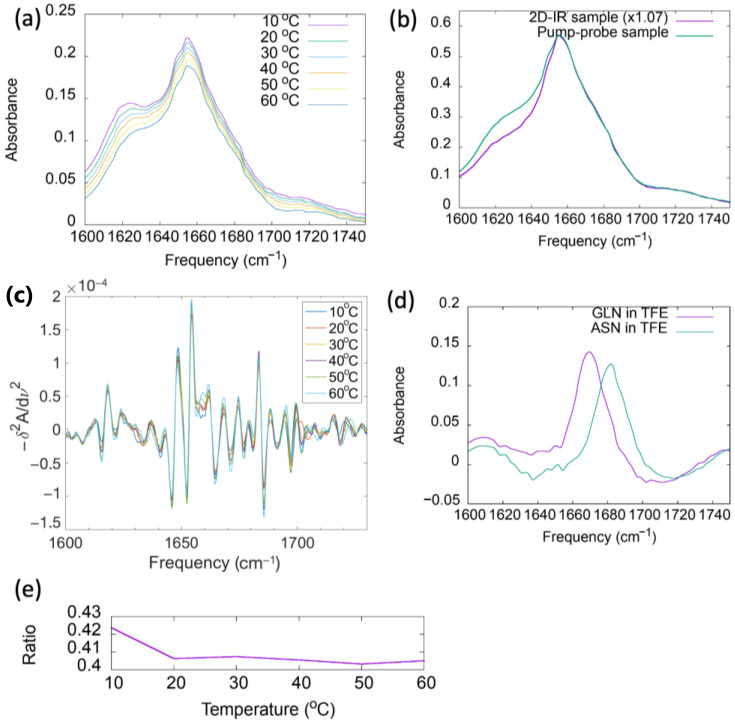
(**a**) Temperature-dependent spectra of the myosin relay helix peptide dissolved in TFE at 8.2 g/L. (**b**) Linear amide I spectra of the peptide dissolved in TFE at 22 g/L. The spectrum corresponding to the pump-probe sample was scaled to match major peak heights for better comparison of line shapes. (**c**) Second derivative plots of the spectra plotted in part (**a**). (**d**) Linear amide I spectra of glutamine and asparagine in TFE dissolved at 9 g/L. (**e**) Ratio of absorbance at 1683 cm^−1^ to absorbance at 1654 cm^−1^ plotted as a function of temperature.

To obtain the temperature-dependent FTIR spectra of the peptide, it was dissolved in TFE at 8.2 g/L. The second derivatives of the spectra are useful for distinguishing overlapping bands [45] and are shown in Figure 3c. The main peak position observed at 1654 cm^−1^ (Figure 3a,c) is characteristic of an α-helical peptide [46]. A nearby peak at 1649 cm^−1^ in the second-derivative spectra (Figure 3c) most likely represents a disordered peptide [47,48,49]. An isosbestic point separates the main peak from a peak at 1618 cm^−1^, lying in the range typical of β-sheet secondary structure, which has the opposite temperature-dependence, indicating conversion between the two types of secondary structure with change in temperature. Confirming the trend seen in the analysis of the CD spectra of the peptide in TFE (Figure 2c), the intensity of the peak in the FTIR spectrum at 1654 cm^−1^, corresponding to α-helical structure, increases with temperature (Figure 3a). That is, lower temperatures favor the peak assigned to β-sheet structure, suggesting that this structure has a lower enthalpy compared to the α-helical peptide. The size of the low-frequency peak was found to vary with the sample (Figure 3b). It was previously observed that solvation in TFE can induce the formation of α-helical structure, which can slowly revert to β-sheet structure [50], and this seems to occur with our peptide. However the room-temperature spectrum of the sample used to obtain Figure 3a practically overlaps with the spectra corresponding to samples prepared at 22 g/L for the 2D-IR and isotropic pump-probe measurements in the frequency range of 1650 cm^−1^ and above (Figure 3b). 

A smaller shoulder peak was also observed at 1683 cm^−1^. The band near 1720 cm^−1^ can be assigned to the carboxylic acid group at the peptide’s C-terminus. Changes in the ratio of absorbance at ~1654 cm^−1^ to that at ~1683 cm^−1^ can be used as an indicator of potential changes in the structure of the α-helical peptide component upon exposure to a laser pulse; thus, the temperature dependence of this ratio was checked to rule out any potential temperature effects. As shown in Figure 3e, this ratio varies from its value of 0.41 at 20 °C by at most 4% over the temperature range studied and is extremely temperature-independent for temperatures higher than 20 °C. 

Since the peptide contains four glutamine residues and three asparagine residues and both residues contain amide groups, the FTIR absorbance of these residues was measured in TFE. Peaks are observed at 1669 cm^−1^ and 1682 cm^−1^ for Gln and Asn, respectively, with molar absorbances only slightly larger in magnitude compared to that of the peptide shoulder at 1683 cm^−1^ (Figure 3d). Despite the fact that the peak frequency of Asn overlaps the peptide band near 1683 cm^−1^, Asn residues account for only 7.7% of the molar mass of the peptide sample and thus contribute little to this band.

### 2.2. Molecular Dynamics Simulations

#### 2.2.1. Hydrogen Bonding

**Figure 4:**
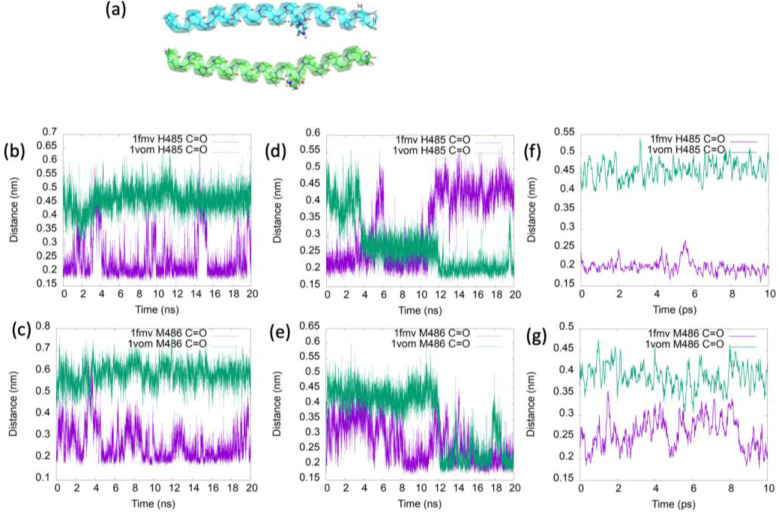
(**a**) Cartoon representations of the two conformers of the myosin relay helix, with H485 shown in ball-and-stick mode. The straight conformer (upper) was extracted from the PDB file 1FMV (apo myosin). The bent conformer (lower) was extracted from 1VOM (ADP-vanadate-bound myosin). In (**b**) and (**c**), hydrogen bond lengths formed by the main-chain H485 and M486 carbonyl groups, respectively, are plotted over 20 ns simulations of the peptide in water. These hydrogen bond distances are plotted for the peptide dissolved in TFE in (**d**,**e**) for 20 ns simulations, and on a 10 ps timescale in (**f**,**g**).

We extracted the coordinates of the two different forms of the myosin relay helix, namely the straight conformer and the bent conformer, from the coordinates of two crystal structures in the Protein Data Bank. In the 1FMV [51] crystal structure, no nucleotide is bound to mysoin, and the relay helix is straight. On the other hand, 1VOM [52] is an ADP-vanadate-bound myosin structure, representative of the ADP-P_i_-bound structure with the bent conformation of the relay helix (Figure 4a). Hereafter, the two structures of the relay helix extracted from these two PDB entries are referred to simply as 1fmv and 1vom. The bend in the relay helix is characterized by breaks in the hydrogen bonding chains forming its α-helical structure. Next, 20 ns molecular dynamics simulations of the two conformers were run, and hydrogen bond distances were calculated along the peptide main chains. Based upon the molecular dynamics simulations, in 1vom, broken hydrogen bonds occur at the position of the bend in the helix. Specifically, we analyzed the trajectories of the straight (1fmv) and bent (1vom) conformers to compare the distances between main-chain carbonyl groups of residues and the main-chain NH groups of the residues four places ahead in the sequence. Hydrogen bonds are most often broken in the 1vom trajectory for the pairs H485/V489 (positions 22/26 in the peptide) and M486/E490 (although the F487/Q491 hydrogen bond is also broken at times). These two hydrogen bond distances are plotted in Figure 4b,c for the two conformers in water and in Figure 4d,g for the peptides in TFE solution. In the case of 1fmv in TFE, one of the hydrogen bonds is periodically broken. Regarding 1vom in TFE, both hydrogen bonds can be formed. For both conformers, these distances are more rigid in water than in TFE over the simulations.

#### 2.2.2. Principal Component Analysis

**Figure 5:**
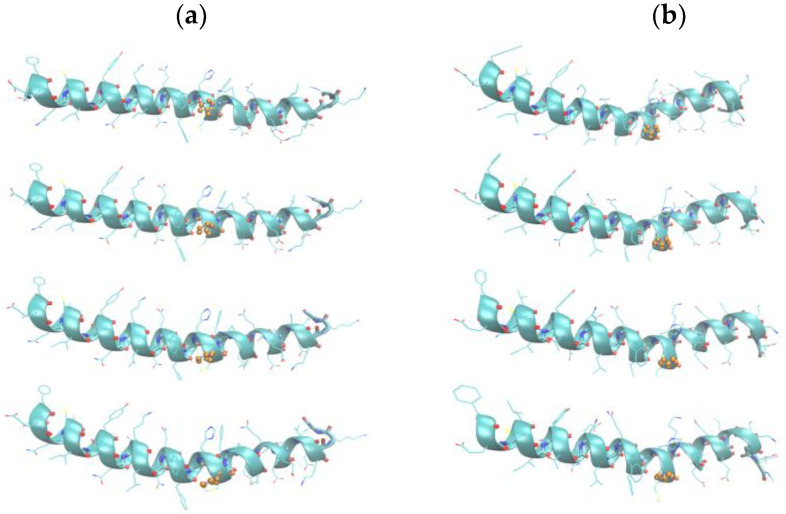
Snapshots showing the range of motion along first principal components for 2 ns trajectories initiated at (**a**) 1fmv and (**b**) 1vom. (H485 is shown in ball-and-stick mode).

The existence of bent and straight conformers of the myosin relay helix suggests that the dynamics of the peptide might show interconversion between the two states. The principal components of the motion of the myosin relay helix were determined from 2 ns molecular dynamics trajectories of the peptide in TFE, using as initial coordinates either the solvent-equilibrated 1fmv or 1vom structure. The first principal components of motion, i.e., the major large-scale, correlated directions of the motion of the peptide’s atomic positions, were then visualized by creating pseudo-trajectory files that show the range of the peptide’s dynamics along these components of the two trajectories. We found that the first principal components of motion correspond to the bending of the helix for the straight conformer (1fmv) and to the straightening of the helix for the bent conformer (1vom) (Figure 5).

#### 2.2.3. Metadynamics Simulations

**Figure 6:**
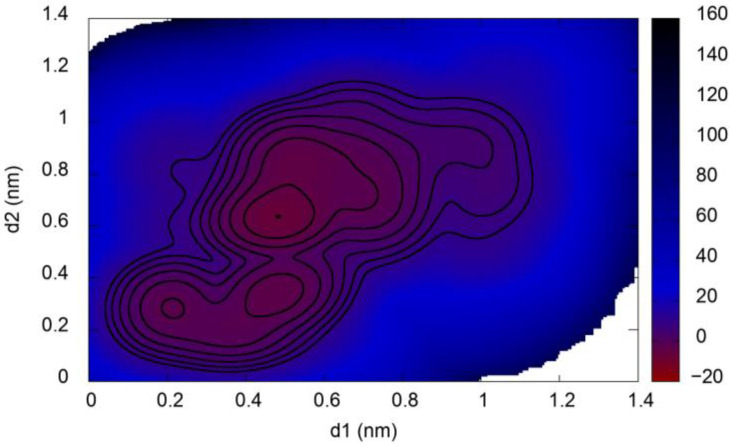
Free energy surface of the myosin relay helix in TFE as a function of the collective variables d1 and d2, defined as the lengths of the hydrogen bonds formed between the main-chain H485 and M486 CO groups and the main-chain NH groups of V489 and E490, respectively. Contours are spaced at intervals of 2.5 kJ/mol from the global free energy minimum.

Metadynamics simulations can be used to determine the free energy surface of a molecular system as a function of user-defined collective variables (CVs). This is achieved by the addition to the force field of a history-dependent bias potential composed of Gaussian functions of the CVs during a molecular dynamics trajectory, which pushes the molecular system to explore regions of high free energy. The equilibrated 1fmv conformer was chosen as the initial configuration for metadynamics simulations. Figure 6 depicts the free energy surface of the myosin relay helix peptide in TFE, where the lengths of hydrogen bonds formed between the main-chain H485 and M486 carbonyl groups and the main-chain NH groups of V489 and E490, respectively, were chosen as CVs, labeled as d1 and d2. The computed free energy surface displays a global minimum of ΔG = −7.54 kJ/mol at (d1, d2) = (0.48 nm, 0.64 nm). There also exist two local minima at (d1, d2) = (0.47 nm, 0.34 nm) and (d1, d2) = (0.21 nm, 0.28 nm), which differ in energy from the global free energy minimum by 3.36 kJ/mol ≈ 1.4 kT and 4.67 kJ/mol ≈ 1.9 kT, respectively, and are separated from it by a kinetic barrier of only ~2 kT. Each of these minima correspond to an ensemble of conformations, which can be generalized as follows. In the second local minimum, where both hydrogen bonds H485-V489 and M486-E490 are maintained, the first 27 or more residues of the 35-residue peptide are strictly helical, and there is always a C-terminal α-helical region as well. For the global minimum and the first local minimum, there is a kink in the helix at these two hydrogen bonds, followed by a partially helical C-terminal portion of the peptide.

The more stable minima at values of d1 and d2 larger than the cutoff value for hydrogen bonding of ~0.35 nm indicate that the bent conformer of the peptide is favored over the straight conformer in TFE. However, the free energy barrier of ~2 kT between the free energy minima corresponding to broken and stabilized hydrogen bonds implies that the hydrogen bonds can easily break and reform.

When the metadynamics simulation was initiated at the 1vom conformer, the computed free energy surface differs somewhat compared to the surface generated with 1fmv as the initial configuration, because of the finite simulation time of 200 ns and the fact that the two chosen CVs do not fully characterize the phase space available to the peptide. However, the computed free energy surface is qualitatively similar to Figure 6. A global free energy minimum is found at (d1, d2) = (0.74 nm, 0.89 nm), which is lower in energy than the two local minima in the free energy surface seen at (d1, d2) = (0.47 nm, 0.43 nm) and (d1, d2) = (0.20 nm, 0.22 nm) by differences of 3.21 kJ/mol ≈ 1.3 kT and 6.01/mol ≈ 2.4 kT, respectively.

#### 2.2.4. Computed Linear Amide I Spectra

**Figure 7:**
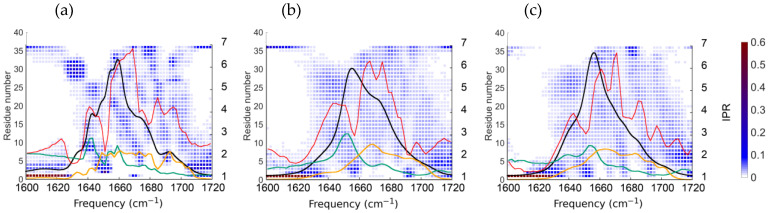
Linear amide I spectra (black lines) of the perfectly helical conformer (**a**), 1fmv (**b**), and 1vom (**c**) computed using 2 ns molecular dynamics simulations. IPR as a function of frequency in red (right-hand *y*-axis), oscillator strength in green, density of states in orange, and squared eigenstate components colored by the scheme on the right are shown superimposed on the spectra.

To identify the spectroscopic signatures of the bent and straight conformations, FTIR spectra were calculated for the bent and straight conformers of the myosin relay helix solvated in TFE, based on 2 ns molecular dynamics simulations. The spectrum for the rigid peptide constrained to a perfectly α-helical conformation was also calculated (Figure 7). The main peak characteristics of α-helical structure are seen in the spectra at 1655 cm^−1^, 1656 cm^−1^, and 1659 cm^−1^, for 1vom, 1fmv, and the perfect α-helix, respectively. A higher frequency peak at 1672 cm^−1^ is observed in the spectrum calculated for 1fmv, but the bent conformer 1vom instead shows a much smaller shoulder at about 1683 cm^−1^, similarly to that seen in the experimental spectrum. The higher frequency peak at 1673 cm^−1^ is also visible in the spectrum of the perfectly α-helical conformer, but not in that of 1vom, substantiating that it results from the straight character of the helix. This is interesting since absorbance at ~1675–1685 cm^−1^ has, by contrast, previously been associated with weakly hydrogen-bonded structures, as for example in antiparallel beta sheets [53] or when hydrogen bonding to water is weakened by the addition of TFE [54]. Note that the spectrum of the perfectly α-helical conformer presents a more jagged appearance than the other two. This is because the fluctuations in the vibrational frequencies of subensembles of amide I groups are dampened by the high restraints placed on the positions of peptide atoms, resulting in smaller homogeneous linewidths of the amide I oscillators.

The time-averaged square magnitudes of the eigenstate components of the system Hamiltonian were computed and are superimposed upon the computed spectra (Figure 7), where they are shown as a function of frequency by the color scheme, along with a plot of inverse participation ratios (IPR) values. We also show the individual contributions to the computed infrared absorption intensity of the peptide from dipole oscillator strengths, ${\left|\mu \right|}^{2}$, and the density of states, respectively. The intensity of the FTIR spectrum is calculated as follows:

(1) $$I\left(\omega \right)=Re\int_{0}^{\infty }dt\;e^{-i\omega t}\sum_{nm}\sum_{j=1}^{3}{\mu_{nj}\left(0\right)F_{nm}\left(t\right)\mu }_{mj}\left(t\right)e^{-t/2T}$$

where the matrix F(t) is the time-evolved vibrational Hamiltonian, T is the amide I lifetime, and ${\vec{\mu }}_{n}$ is the transition dipole moment of the $n^{th}$ amide I eigenstate [55]. The intensity may be approximated as follows:

(2) $$\;I\left(\omega \right)=\langle \sum_{n}\Delta \left(E-E_{n}\right){\left\|{\vec{\mu }}_{n}\right\|}^{2}\rangle ,\;\;$$

where $E_{n}$ is the energy of the $n^{th}$ amide I eigenstate [56]. Therefore, it may be seen that the intensity of the spectrum depends not only on the oscillator strength but also on the density of states corresponding to each frequency. The peptide has 36 vibrational eigenstates corresponding to the number of amide I oscillators. The association of these eigenstates with larger oscillator strengths depends upon their components in the basis of the 36 local amide I oscillators tending to point in the same direction as one another rather than in opposing directions.

The relative dipole strengths of the two peaks differ depending upon the peptide’s conformation. In the case of either the straight conformer 1fmv or the perfect α-helix, the dipole strength of the major peak is 2.7 times that of the minor peak, while for the bent conformer, 1vom, the dipole strength of the major peak is 4 times that of the minor shoulder. In the case of 1fmv, the minor peak corresponds to a higher density of states than the major peak, and for 1vom and the perfect α-helix, the density of states is roughly the same at both positions. Thus, in all cases, the cause for the higher absorbance at the major peak is its higher associated oscillator strength.

### 2.3. 2D-IR Spectra

#### 2.3.1. Experimental 2D-IR Spectra

**Figure 8:**
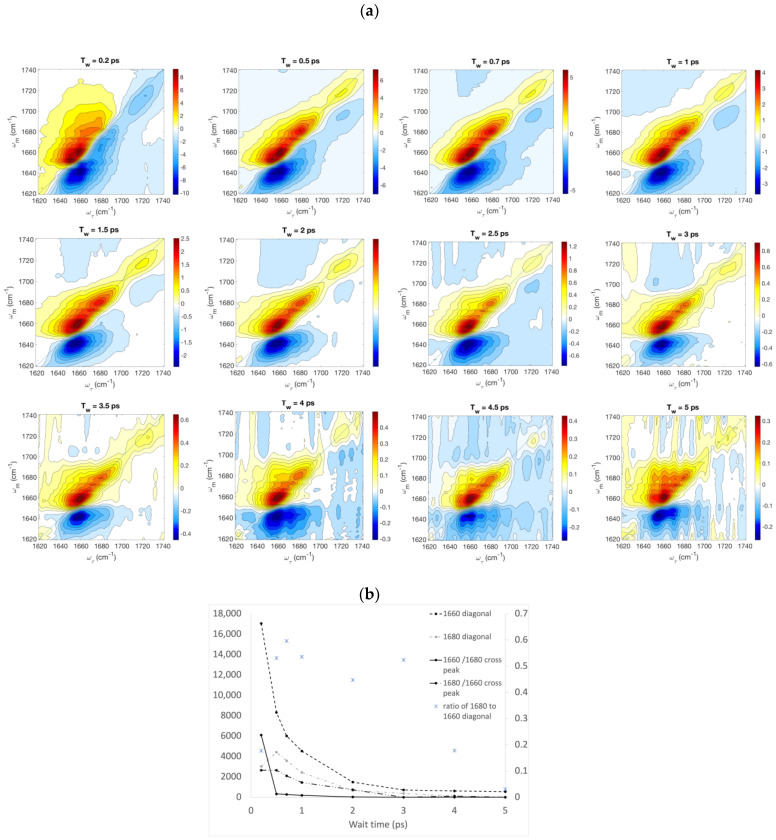
(**a**) 2D-IR amide I spectra of the myosin relay helix peptide dissolved in TFE, at wait times varying from 0.2 to 5 ps, as a function of the time evolution frequency, ω_τ_, and the detection frequency at the monochromator, ω_m_. (**b**) Volumes of Gaussian fits to diagonal peaks and cross peaks and ratios of diagonal peak volumes (right-hand *y*-axis), plotted as a function of wait time.

We measured the 2D-IR spectra of the peptide dissolved in TFE at a concentration of 22 g/L. Pump-probe data were simultaneously collected to perform automatic phasing. In the FTIR spectrum corresponding to this sample, the ratio of the peak height at 1654 cm^−1^ to that at 1683 cm^−1^ is 2.31, and the inverse ratio is 0.43 (Figure 3b). The 2D-IR signal is shown at various time delays between the second and third pulses, ranging from 0.2 ps to 5.0 ps, in Figure 8. The red (positive) diagonal peaks near 1660 cm^−1^ and 1680 cm^−1^ are the fundamental peaks corresponding to stimulated emission by the third pulse, following creation of a coherence by the first pulse and transition from the ground to the first excited state stimulated by the second pulse. These positive fundamental peaks also correspond to ground-state depletion by the second pulse reducing the number of transitions to the first excited state by the third pulse. Blue (negative) peaks just below the diagonal correspond to excitations by the third pulse from the first to the second excited vibrational state and are offset from the fundamental peaks along the probe axis by the anharmonic shift. The presence of cross-peaks demonstrates that the populations at 1660 cm^−1^ and 1680 cm^−1^ are coupled with one another. The diagonal and off-diagonal peaks at a frequency near 1720 cm^−1^ correspond to the C-terminal carboxy group.

Volume fitting of the 2D-IR peaks was used to verify the presence of cross-peaks in the spectra and to evaluate the corresponding intensities (Table 1 and Figure 8b). The residuals obtained both with and without cross-peaks are shown in Figure S2a–h, and the fitting parameters are listed in Table S1. The lower residuals when volumes were fit with cross-peaks, compared to when fit without cross-peaks, confirmed the existence of cross-peaks at time delays between 0.2 and 2 ps. We note that, for wait times between 0.5 and 2 ps, the residual is specifically reduced in the area where cross-peaks would be observed, when fitting with cross-peaks compared to fitting without them (Figure S2b–e). The existence of cross-peaks signifies vibrational energy transfer between local vibrational modes, and/or chemical exchange, i.e., a change in the molecular conformation between the times of the pump and the probe (second and third) pulses. Vibrational coupling can lead to vibrational energy transfer both by nonadiabatic hopping between different vibrational eigenstates and adiabatically as changes in the contributions of individual local modes to vibrational eigenstates are induced by fluctuations in the solvating environment [26,57]. Because the cross-peaks are not well resolved and can be attributed to multiple causes, we avoid conclusions about their time evolution.

#### 2.3.2. Computed 2D-IR Spectra

**Figure 9:**
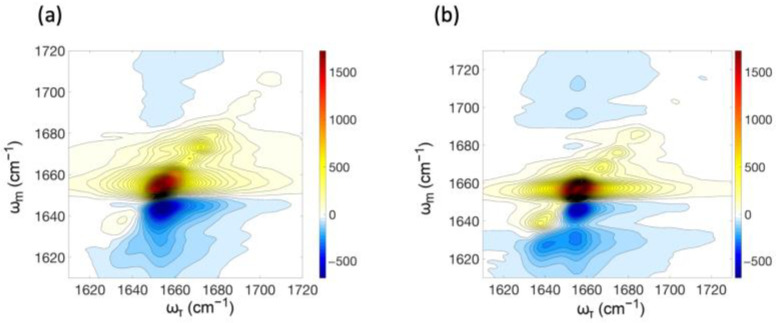
2D-IR amide I spectra computed from 2 ns simulations of (**a**) 1fmv and (**b**)1vom in TFE solvent.

We wanted to know whether the equilibrium between the bent and straight conformers of the myosin relay helix is influenced by the laser pulse. To address this issue of laser-induced chemical exchange, 2D-IR spectra were computed for the 1fmv and 1vom conformers, corresponding to a wait time of 0 ps, with no influence of the laser pulse on the peptide conformation included in the modeling (Figure 9). These spectra were fit by the same volume fitting program as was used to obtain the values in Table 1. Residuals are shown in Figure S2i,j and the fitting parameters are listed in Table S2. In the case of 1fmv, the resulting diagonal peaks were found to have volumes of 3351 for the 1660 cm^−1^ diagonal peak, 343 for the 1680 cm^−1^ diagonal peak, 0 for the 1660 cm^−1^/1680 cm^−1^ cross-peak, and 2770.4 for the 1680 cm^−1^/1660 cm^−1^ cross-peak. For 1vom, these values are 2775 (1660 cm^−1^ diagonal peak), 27 (1680 cm^−1^ diagonal peak) 0.0 (1660 cm^−1^/1680 cm^−1^ cross-peak) and 2611 (1680 cm^−1^/1660 cm^−1^ cross-peak). The cross-peaks seen in the computed spectra illustrate that the vibrational energy transfer between coupled amide I modes at least partially accounts for the presence of cross-peaks in our experimental spectra, since the wait time used in computing these was zero and thus no chemical exchange was possible. 

There are notable differences in the ratios of the magnitude of the volume of the diagonal peak at 1680 cm^−1^ compared to that of the major diagonal peak at 1660 cm^−1^ between the computed and experimental spectra at wait times between 0.5 ps and 1 ps. During this range of time delays, the ratio of these two volumes is consistently greater than 0.5 in the experimental data, reaching a maximum value of 0.6 at 0.7 ps (Figure 8b), but it is 0.1 for 1fmv and 0.01 for 1vom in the computed spectra. In this regard, the experimental spectra bear more resemblance to the 2D-IR spectrum computed for the straight conformer 1fmv than that computed for 1vom, while on the other hand, the linear experimental spectra were found to be more similar to the computed spectra for the bent conformation. 

A conformational change caused by a pulse with a frequency of 1680 cm^−1^ might increase the size of the fundamental peak at 1680 cm^−1^ since the dipole strength of the mode at this frequency might become stronger because of the conformational change, or conversely, a pulse with a frequency of 1660 cm^−1^ might decrease the relative size of the fundamental peak at 1660 cm^−1^. Our hypothesis that excited vibrational modes can prevent broken hydrogen bonds at H485/M486 and V489/E490 (positions 22/26 and 23/27 in the peptide) raises open questions about the details of such a mechanism. A vibrational amide I mode can exert a direct force on a hydrogen bond only if it is located there. In Figure 7b,c, the average eigenstate components at the carbonyl groups of residues 22 and 23 are small for both the bent and straight conformers at the frequencies 1660 cm^−1^ and 1680 cm^−1^, and so the mechanism remains unclear to us. 

### 2.4. Pump-Probe Transient Absorption Spectra

**Figure 10:**
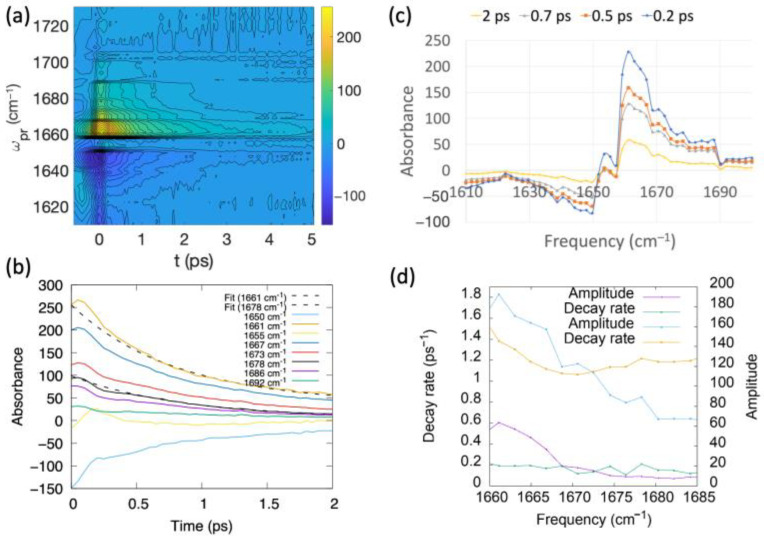
(**a**) Contour plot of pump-probe transient absorption spectrum of the myosin relay helix peptide, with time delay plotted on the *x*-axis and probe frequency ω_pr_ on the *y*-axis. (**b**) Cross-sections of the pump-probe spectrum for different frequencies, with bi-exponential fits shown to the curves corresponding to frequencies of 1661 cm^−1^ and 1678 cm^−1^, respectively. (**c**) Cross-sections of the pump-probe spectrum at time delays of 0.2, 0.5, 0.7, and 2 ps. (**d**) Decay rates and amplitudes of bi-exponential fits of cross-sections of the pump-probe spectra as a function of frequency.

The pump-probe isotropic transient absorption spectrum of the myosin relay helix peptide at 22 g/L is plotted as a function of time delay in Figure 10a. Negative absorption is used here to denote new absorption induced by the probe and corresponds to excited state absorption. Figure 10b shows the decay in time of the pump-probe signal at frequencies ranging from 1650 cm^−1^ to 1691 cm^−1^. Note that the overlap between the pump and probe pulses results in a non-zero absorbance value at a time delay of 0. 

Fits to double exponential decay curves of the time-dependent decay of pump-probe signals at times ranging from 0.5 to 10 ps are plotted in Figure 10b for the frequencies corresponding to the two peak maxima at 1661 cm^−1^ and 1678 cm^−1^. Decay rates and amplitudes from such fits are plotted as a function of frequency, ranging from 1660 cm^−1^ to 1685 cm^−1^, in Figure 10d. Decay rates vary little over this range of frequencies. The decay rates are 1.38 ps^−1^ at 1661 cm^−1^ and 1.22 ps^−1^ at 1678 cm^−1^. In Figure 10c, slices of the pump-probe data are shown at time delays of 0.2 ps, 0.5 ps, 0.7 ps, and 2 ps. The pump-probe cross-sections seen in Figure 10c should be close in value to the integral over the frequency ω_τ_ of the 2D-IR plots with the corresponding wait times. The frequency of the major peak is blue-shifted from 1654 cm^−1^ in the linear FTIR spectrum to 1661 cm^−1^ in the pump-probe spectra. A smaller peak may also be observed at 1678 cm^−1^, close to the position of the peak at 1683 cm^−1^ in the linear spectrum. Additionally, there is another peak in the pump-probe spectra at 1671 cm^−1^. This position almost matches the position of the higher frequency peak seen in the computed linear spectrum for the straight 1fmv conformer at 1672 cm^−1^. The ratio of the peak height at 1661 cm^−1^ to that at 1678 cm^−1^ is 3.1 at 0.2 ps. This ratio is 2.8 at 0.5 ps and 2.7 at 0.7 ps, demonstrating a decrease in the ratio with time from 0.2 to 0.7 ps, and it then increases to 2.9 at 1 ps and 3.9 at 2 ps. Absorbance is proportional to ${\left|\mu \right|}^{4}$ in a pump-probe signal and to ${\left|\mu \right|}^{2}$ in a linear spectrum. Thus, the value at 0.2 ps is consistent with the ratio of the absorbance at 1661 cm^−1^ to that at 1678 cm^−1^, corresponding to the linear spectrum shown in Figure 3b of 1.75. That is, if it were entirely due to oscillator strength (but not to the density of states), as indicated by the simulations, this value should be the square of the value observed for the linear spectrum. These observations could potentially signify a conformational change in the peptide brought about by the pump-pulse at time delays from 0.5 to 1 ps.

## 3. Discussion

Because the degree of helicity of the myosin relay helix is significant to large-scale conformational changes in myosin associated with its chemomechanical cycle, we have measured and analyzed the amide I vibrational spectra of the peptide corresponding to this helix. Using computer simulations, we have shown that, for two of the main-chain carbonyl oxygen atoms, distances to the amino hydrogen atom three residues ahead in the sequence can take on values corresponding to either broken or stabilized hydrogen bonds. Interestingly, Sataric et al. [58] previously put forward a crude estimate of about 4 kT for the energy difference between the two conformational states of the myosin relay helix and used this value to support the possible involvement of the helix in energy transfer during the power stroke. The present work indicates that the two states are separated by only about 2 kT, supporting the possibility that system relaxation in the presence of an amide I excitation might be able to elicit the formation of these hydrogen bonds. Experimental 2D-IR and pump-probe infrared spectra revealed that an amide I excitation does not have a long-lasting effect on the myosin relay helix peptide conformation. However the pump-probe spectrum showed a small increase in the relative peak height at 1678 cm^−1^ compared to the main peak at 0.5–1 ps and, moreover, the volumes of the fundamental peak at ~1680 cm^−1^ in the 2D-IR spectra were relatively high at wait times of 0.5 to 2 ps, which might be indicative of a conformational change toward the straight conformer on the ps timescale. As seen in Figure 4f,g, the hydrogen bonds of the α-helical main-chain break and reform on the timescale of several picoseconds. In reference [25], molecular dynamics simulations were performed that modeled the effect of a quantum vibrational excitation on the dynamics of a peptide α-helix. In addition to the classical forces, the simulations included forces on hydrogen-bonded main-chain atoms given by the gradient with respect to the atomic positions of the Davydov Hamiltonian describing the interaction between the quantum vibration and the classical molecular system. Although the effect was minimal in the simulations, the vibrational excitation produced a force on the hydrogen-bonded main-chain atoms of an α-helix proportional to the hydrogen bond length, changing these lengths. Thus, it is possible that the presence of a vibrational excitation may reduce the timescale for the configurational change related to the stabilization of hydrogen bonds and the associated straightening of the helix from several picoseconds to less than a picosecond.

The fact that the energy released during ATP hydrolysis is small and short-lived has caused uncertainty about how this energy might make a dent on large-scale protein dynamics. However, couplings between conformational changes in proteins and vibrational excitations have previously been observed [59,60], such as, for example, low-frequency bending motions coupled to the stretch of a CO ligand bound to a de novo metalloenzyme observed by 2D-IR [59]. Based on the present work, it seems possible that an amide I excitation might directly contribute to the activation of an important conformational change in the myosin relay helix or to the stabilization of the peptide against a conformational change during ATP hydrolysis in myosin. That is, together with other conformation-biasing factors related to the change in the nucleotide phosphorylation state of myosin, the effect of an amide I excitation might help to impact protein conformation. Further experimental studies of the myosin relay helix are warranted to test this hypothesis, such as, for example, examination of its dynamics by time-resolved step-scan FTIR following triggering by a laser pulse.

In our adaptation of the Davydov soliton model, straightening of the relay helix is proposed to be an early step in the power stroke that is initiated within picoseconds of ATP hydrolysis by energy transfer from the vibrationally excited orthophosphate molecule to the relay helix. Whether orthophosphate release from the active site occurs before or after the power stroke is a highly debated issue [61,62]. It has been determined experimentally by Förster resonance energy transfer (FRET) studies that the timescale for orthophosphate release ranges from 3 to 50 ms and that the timescale for the power stroke is 2–3 ms [61]. FRET data could be fit by a kinetic simulation model assuming that the power stroke occurred before orthophosphate release into solution, but not if orthophosphate was assumed to be released first [63]. Moreover, blocking orthophosphate from reaching the exit tunnel with the mutation S217A or exposure to high concentrations of orthophosphate affected neither the size nor the rate of the power stroke, implying that the power stroke occurs before orthophosphate enters the exit tunnel [64]. However kinetic models showed that the timing of orthophosphate release from the active state after the power stroke was not consistent with experimental observations of the [Pi]-independence of maximal shortening velocity and monotonous decrease in isometric force with increased [Pi] [62,65]. This led to the multi-step orthophosphate release kinetic model, which predicts that orthophosphate leaves the active site but is trapped at an intermediate secondary binding site before completion of the power-stroke [65]. In support of the multi-step release model, Ma et al. [66] used molecular dynamics simulations to examine shifts in the conformational states of the myosin motor domain in different nucleotide-bound states or in the apo-state and concluded that orthophosphate release from the active site gates the lever arm swing. In summary, a likely sequence of events might be as follows: First ATP is hydrolyzed, creating a vibrationally excited orthophosphate molecule that binds to a secondary binding site; then, picosecond timescale vibrational energy transfer causes a small conformational change in the relay helix, which due to phosphate release from the active site is amplified to the rotation of the lever arm on a millisecond timescale; and later, orthophosphate is released into solution on a timescale of tens of milliseconds. 

A better understanding of how the energy released upon ATP hydrolysis in myosin is coupled to the power stroke may have implications for mechanisms of energy flow in other protein NTPases. For example, in actin, ATP hydrolysis and orthophosphate release promote spontaneous depolymerization and strengthen the binding to actin of proteins that accelerate depolymerization. A recent study found that the global conformations of ATP- and ADP-bound actin are the same [67]. It was concluded that orthophosphate release increases overall fluctuations of actin, and this affects conformations of the intra-subunit binding sites including the D-loop [67], which equilibrates between different conformational states in F-actin, including α-helical, disordered, and hairpin configurations [68]. In actin and/or other protein ATPases, vibrational excitations created immediately after ATP hydrolysis may influence the conformation of variable structures, such as the D-loop, in an analogous manner to what we propose occurs within the myosin relay helix, and may in this way impact protein function.

## 4. Materials and Methods

### 4.1. Standard Molecular Dynamics Simulations

Peptide coordinates for the myosin relay helix were extracted from the PDB entries 1VOM [52] and 1FMV [51], respectively, for residues 465–499 of the Dictyostelium myosin II protein. Simulations of each of these conformers in an aqueous environment were performed under periodic boundary conditions using the pmemd.MPI module of AMBER16 [69]. The solvating water was first relaxed, followed by minimization of the entire system. The system was then heated to a final temperature of 300 K at constant volume over a period of 50 ps using a Langevin thermostat, while all solute atoms were restrained by a force constant of 25 kcal/mol. Restraints on solute atoms were next gradually relaxed to zero at constant pressure over 100 ps using a time step of 2 fs, and then a 2 ps equilibration was performed at constant pressure. Finally, the molecular dynamics trajectory was run for 20 ns with a 2 fs time step.

RESP atomic charges for TFE were downloaded from the R.E.D. database [70,71]. The Packmol program [72] was used to make a box with dimensions 24 × 24 × 24 Å containing 125 TFE molecules as follows. Molecules were initially separated using a tolerance of 2.0 Å, and AMBER16 input parameters were created using tleap. A minimization was performed in AMBER16, followed by heating to 300 K at constant volume over a period of 0.1 ns using a 2 fs timestep, and then by equilibration at constant pressure for 0.1 ns using a 2 fs time step. The resulting solvent box was saved as a library in tleap and used to solvate the peptide under periodic boundary conditions. Molecular dynamics simulations in TFE solvent were then performed under similar conditions as those performed in aqueous solution, as described above.

For simulations of the myosin relay helix in the conformation of a perfect α-helix, all phi/psi (φ/ψ) dihedral angle pairs were set equal to −57.8°/−47.0°, using the Gaussview program [73]. Minimization and heating were performed as described above, and then a 2 ps equilibration was run, followed by a 2 ns molecular dynamics simulation of this structure with a force constant of 25 kcal/mol applied to restrain all peptide coordinates.

### 4.2. Metadynamics

Metadynamics simulations were performed using the PLUMED program [74,75,76,77] as a plugin to the AMBER16 [69] molecular dynamics program. The well-tempered metadynamics method was used, which avoids large fluctuations of calculated free energies as the system is pushed to explore the high-energy space by multiplying the heights of Gaussian functions by a weighting factor that is decreased as a phase space region is explored. Gaussian functions were deposited every 500 steps with a height of 1.2 kJ/mol. The Gaussian width was chosen to be 0.05 nm based on the fluctuation of the hydrogen bonding distances in the unbiased simulation. The bias factor was set to 6.0. The simulations were run for 200 ns with a time step of 2 fs, and collective variables were printed every 10 steps.

### 4.3. Principal Component Analysis

The first principal components of motion were determined for 2 ns trajectories initiated at the 1fmv or the 1vom structure, respectively. The first principal component of motion gives the direction of the largest variance in the coordinate space of the atomic positions. A root-mean-square fit of heavy atoms to the first frame was performed and an average frame was determined in cpptraj [78], frames were refit to this average structure, and finally the covariance matrix of atomic coordinates was determined and diagonalized to give the principal components. The original trajectories were projected onto the principal components of motion to obtain pseudo-trajectories, which were visualized in VMD [79]. The range of motion depicted in the pseudo-trajectory was determined from the minimum and maximum values of the projections onto the principal components [80,81].

### 4.4. Experimental Spectra

The peptide with sequence SFEQLCINYTNEKLQQFFNHHMFKVEQEEYLKEKI, comprised of 35 residues and capped by an acetyl group at its N-terminus and a carboxylic acid group at its C terminus, was synthesized at greater than 95% purity by GL Biochem, Shanghai, China. This peptide corresponded to residues 465–499 of myosin (dictyostellium myosin II), which is known as the relay helix.

UV circular dichroism (CD) spectra were measured at temperatures ranging from 10 °C to 70 °C on a J-815 CD spectrometer (Jasco, Tokyo, Japan) with temperature maintained by a PTC-423S/15 temperature controller (Jasco, Tokyo, Japan). The frequency resolution was 0.5 nm and the path length was 0.2 mm. The scanning speed was 100 nm/min, and averaging was performed over 8 accumulations. The existence of two minima at 222 nm and 208 nm in the CD spectrum is characteristic of α-helical structure. The reading at 222 nm can be used to quantify the α-helical content [82]. On the other hand, both α-helices and random coils have the same CD values at 208 nm [82]. As derived by Baldwin and coworkers [32,33], the helical fraction, *f_H_*, can be estimated from the molar ellipticity per residue at 222 nm, [θ]_222_, as given by:

(3) $${\;f}_{H}=\frac{{\left[\theta \right]}_{222}-{\left[\theta \right]}_{C}}{{\left[\theta \right]}_{222}-{\left[\theta \right]}_{H}}$$

where ${\left[\theta \right]}_{C}=2220-53T$ and ${\left[\theta \right]}_{H}=\left(-44000+250T\right)\left(1-\frac{3}{N_{r}}\right)$ are the molar ellipticities per residue of random coil and perfect α-helix, respectively, $N_{r}$ is the number of residues in the peptide, and T is the temperature in °C.

FTIR spectra were measured using a Frontier spectrometer (PerkinElmer, Waltham, MA, USA). The frequency resolution was 0.2 cm^−1^. Samples were placed in 0.45 μm filter tubes and centrifuged at 8000 rpm for 4 min in a 5430R centrifuge (Eppendorf, Hamburg, Germany). During temperature-controlled experiments, filtrates were placed in a temperature-controlled demountable liquid IR cell (Harrick Scientific Products, Pleasantville, NY, USA), which was heated or cooled by circulating water through the collar around the cell, between two 3 mm thick CaF_2_ windows. For room temperature experiments, a DLC-M25 liquid cell was used (Harrick Scientific Products, Pleasantville, NY, USA). CaF_2_ windows were separated using a Teflon spacer to create a path length of 56 μm. The spectrum of pure TFE was calculated separately and subtracted as the background. When measuring the FTIR spectra of asparagine and glutamine, the amino acids were first dissolved in HCl, and then TFE was added to create 9 g/L solutions in 5% by volume HCl/TFE. 

For 2D-IR measurements, the sample was placed between two 2 mm thick CaF_2_ windows with a 56 μm spacer. A one-box Ti:sapphire oscillator (Maitai, Spectra-Physics, Milpitas, CA, USA) and a 1 KHz regenerative amplifier (Solstice Ace, Spectra-Physics, Milpitas, CA, USA) were used to generate an 800 nm pulse, which produced a near-IR pulse by an optical parametric amplifier (TOPAS-prime plus, Spectra-Physics, Milpitas, CA, USA). A mid-IR pulse centered at 1655 cm^−1^ was generated by difference frequency generation and split into three excitation pulses that successively interacted with the sample. The three pulses were focused into the sample in boxcar geometry and then combined with the local oscillator beam. The beams were characterized by three time delays, namely the evolution time t_1_ between the first two pulses, the waiting time T_w_ between the second and third pulses, and the detection time t_3_ between the third pulse and overlap with the local oscillator. The heterodyne-detected signal was dispersed in a monochromator (iHR 320, Horiba, Kyoto, Japan) and measured using a 2 × 64-element MCT (HgCdTe) array detector (Infrared Systems Development Corporation, Winter Park, FL, USA). The signal was Fourier-transformed over t_1_ and t_3_ to give the 2D spectrum as a function of the frequencies ω_1_ and ω_3_, alternatively denoted as ω_τ_ and ω_m_, respectively. The rephasing and non-rephasing spectra were summed to give the purely absorptive 2D-IR spectrum, and separate plots were created as a function of ω_τ_ and ω_m_ for different values of T_w_ in MATLAB ver. R2018b. 

Diagonal and cross-peak volumes seen in snapshots of 2D-IR spectra at different waiting times were fitted to tilted 2-dimensional Gaussian functions using an in-house code in MATLAB ver. R2019a [83]. The tilt angles used were 30° for diagonal peaks and 0° for cross-peaks, based upon best fit. Fundamental peak amplitudes were searched in the range of 0–50, starting at 10; cross-peak amplitudes were scanned in the range of 0–6, starting at 2; ω_τ_ peak centers were scanned in the range of 1655–1670 cm^−1^, starting at 1660 cm^−1^; ω_m_ peak centers were scanned in the range of 1660–1685 cm^−1^, starting at 1680 cm^−1^; all peak widths at the lower frequency (1660 cm^−1^) in both the ω_τ_ and the ω_m_ directions started at 6 cm^−1^ and peak widths at the higher frequency (1680 cm^−1^) started at 5 cm^−1^ and were scanned from 0 to 13 cm^−1^, except for the fundamental peak near 1660 cm^−1^, for which the width was scanned from 0 to 20 cm^−1^; and all anharmonicities were scanned from 10–20 cm^−1^, starting at 15 cm^−1^.

IR pump-probe experiments used a mid-IR pulse generated by an OPA in the laser set-up described above, which was split by a ZnSe beam splitter into pump and probe pulses with a 9:1 intensity ratio. The pump and probe beams were focused onto the sample and the resulting signal was frequency-resolved with a monochromator and measured by an MCT array detector. Pump-probe spectra were obtained for two purposes; firstly, for phasing of 2D-IR signals by an in-house MATLAB script, and secondly, for determining population relaxation. To determine population relaxation, the polarization of the probe beam with respect to the pump beam was controlled using a rotating polarizer. Measurements were taken for both the parallel (S_||_) and perpendicular (S_⊥_) relative orientations. The isotropic time-dependent signal representing population relaxation was then determined as follows [84]:

(4) $$P\left(t\right)=\frac{{S_{∥}\left(t\right)+2*S}_{⊥}\left(t\right)}{3}.$$

Bi-exponential fits of frequency cross-sections of these isotropic transient absorption signals were applied to the following form:

(5) $$P\left(t\right)=A_{1}e^{-B_{1}t}+A_{2}e^{-B_{2}t},$$

where *A*_1_, *A*_2_, and *B*_1_, *B*_2_ define the amplitudes and decay rates, respectively. Fits were performed in *gnuplot* using all data points with time delays greater than 0.5 ps.

### 4.5. Computed Spectra

The linear amide I spectra of the myosin relay helix peptide were calculated using the *g_amide* program [85,86] with the DC15 map [87,88,89], and the *g_spec* program, both freely available on GitHub [90,91]. Since *g_amide* requires as input a Gromacs [92,93] molecular dynamics trajectory and topology file, AMBER topology files and 2 ns trajectories, computed as described above, were converted to Gromacs format using the *cpptraj* [78], *acpype* [94], and *catdcd* [95] programs. The *g_amide* program employs a set of maps parameterized as a function of the local electrostatic environment and dihedral-angles to calculate amide I local mode frequencies [96], transition dipole moments [97], and couplings between amide groups [87] at all frames of the trajectory and then uses these to construct an exciton-like Hamiltonian. 

Calculated Hamiltonian matrices and transition dipole moments obtained from *g_amide* were input to the *g_spec* program to predict the linear and 2D amide I spectra. When calculating the 2D amide I spectra, numerical integration of the Schrodinger equation (NISE) method was used to evolve the excitonic wavefunction along the system trajectory [98]. A perturbative approximation was used to determine the two-quantum states rather than diagonalizing the two-quantum Hamiltonian.

The *g_spec* program was modified to bin computed eigenmodes according to frequency and, in this way, to calculate the site components of eigenmodes, inverse participation ratios (IPRs), and oscillator strength as a function of frequency. IPR was computed as follows:

(6) IPR=1∑n=1Nφjn4,

where N=36 is the number of amide I oscillators, and $\varphi_{jn}$ are the site components of eigenmode *j*. IPR values can range from 1, when an excitation is localized on a single oscillator, to N, when an excitation is completely delocalized.

When fitting the computational 2D-IR data to a Gaussian fit, the input file that specified the parameter space to be searched in the Gaussian fit was only slightly different from that used for the experimental fit data, since they were adjusted to obtain better fits corresponding to the datasets. Fundamental peak amplitudes were searched in the range of 0–20, starting at 5 (lower frequency peak), or in the range of 0–10, starting at 2 (higher frequency peak); cross-peak amplitudes were scanned in the range of 0–6, starting at 2; ω_τ_ peak centers were scanned in the range of 1650–1660 cm^−1^, starting at 1655 cm^−1^; ω_m_ peak centers were scanned in the range of 1670–1680 cm^−1^, starting at 1675 cm^−1^; all peak widths at the lower frequency (1655 cm^−1^) in both the ω_τ_ and the ω_m_ directions started at 6 cm^−1^ and peak widths at the higher frequency (1675 cm^−1^) started at 5 cm^−1^ and were scanned from 0 to 10 cm^−1^, except for the fundamental peak near 1655 cm^−1^, for which the width was scanned from 2 to 20 cm^−1^; and all anharmonicities were scanned from 8–15 cm^−1^, starting at 10 cm^−1^.

## Figures and Tables

**Table 1:** Volumes corresponding to Gaussian fit of peaks in the 2D-IR spectra of the myosin relay helix peptide. Wait times are given in units of ps.

Title 1	T_w_	1660 cm^−1^ Diagonal	1680 cm^−1^ Diagonal	1660 cm^−1^/1680 cm^−1^ Cross-Peak	1680 cm^−1^/1660 cm^−1^ Cross-Peak	Residual
cross-peaks	0.2	17,049	3024	6095	2648	3133
no cross-peaks	0.2	24,535	9657			3661
cross-peaks	0.5	8324	4420	321	2645	1133
no cross-peaks	0.5	8395	3299			1449
cross-peaks	0.7	6010	3582	274	2094	693
no cross-peaks	0.7	6674	2654			915
cross-peaks	1	4532	2427	186	1443	355
no cross-peaks	1	5049	1804			463
cross-peaks	2	1489	666	30	736	69
no cross-peaks	2	1662	850			87
cross-peaks	3	712	373	0	0	25
no cross-peaks	3	676	371			25
cross-peaks	4	616	110	5	113	9
no cross-peaks	4	1329	80			10
cross-peaks	5	549	17	0	0	5
no cross-peaks	5	987	9			5

## Data Availability

The following files are available free of charge by request: Experimental UV circular dichroism and linear amide I spectra, molecular dynamics simulation trajectories, computed and experimental 2D-IR spectra, and experimental pump-probe spectra.

## Supplementary Materials

Supporting information can be downloaded at: https://www.mdpi.com/article/10.3390/ijms25126406/s1.
