# Supplementary material for: Study of the Myosin Relay Helix Peptide by Molecular Dynamics Simulations, Pump-Probe and 2D Infrared Spectroscopy
Source: Int J Mol Sci. 2024 Jun 10;25(12):6406. doi: 10.3390/ijms25126406 (PMC11203622; doi:10.3390/ijms25126406)
Supplement: Supplementary file 1 [file ijms-25-06406-s001.zip › ijms-2957675-supplementary.pdf]

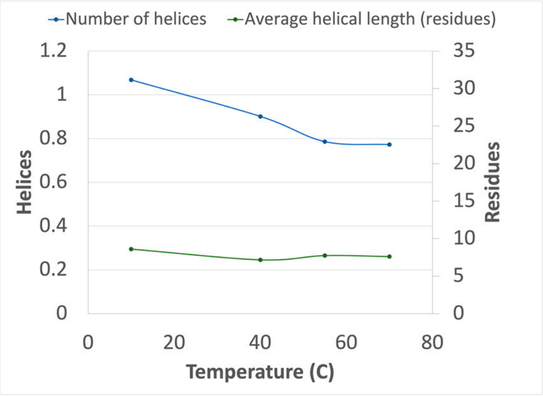

Figure S1. Number of helices and average helical length of the myosin relay helix peptide as a function of temperature in water.

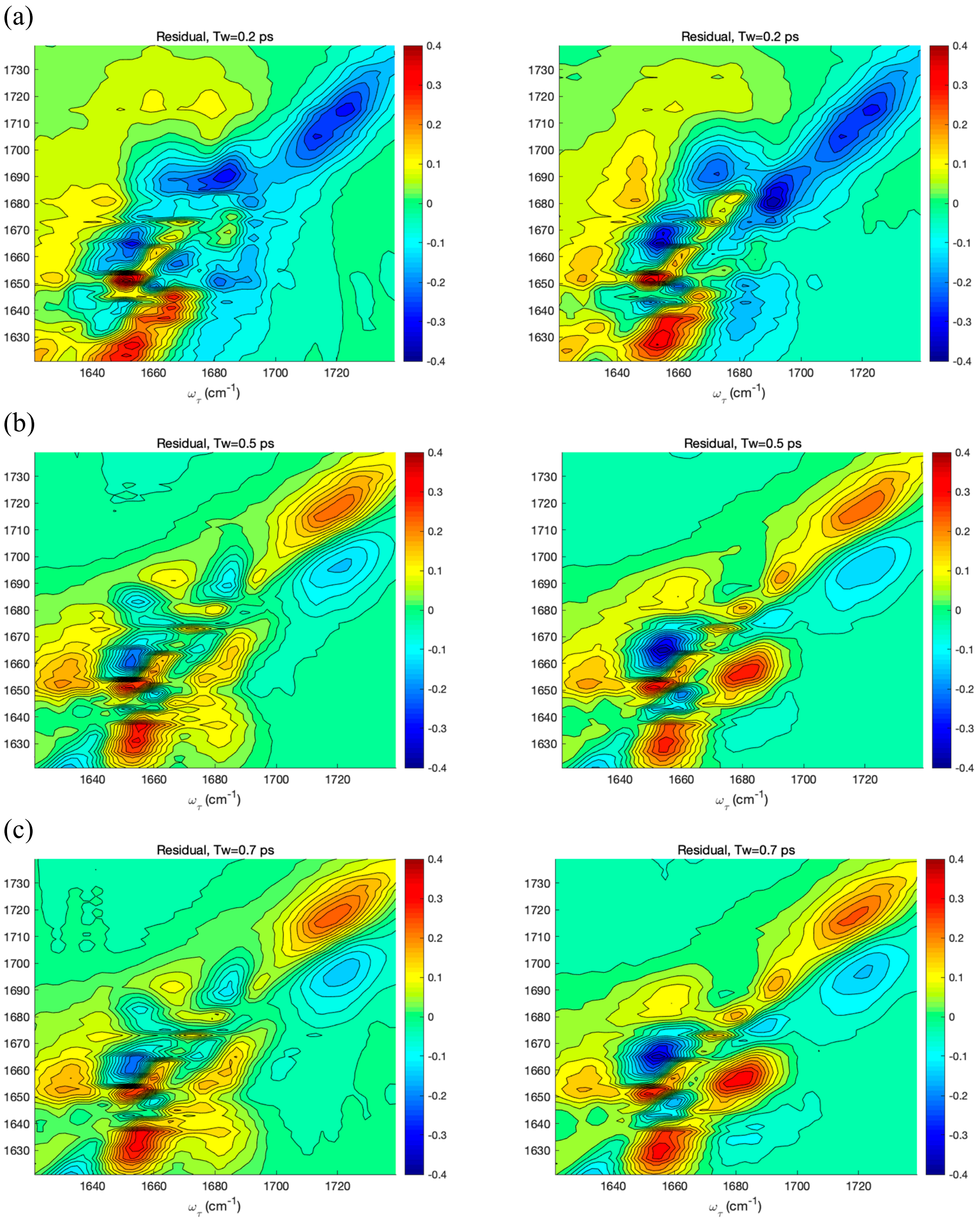

(d)

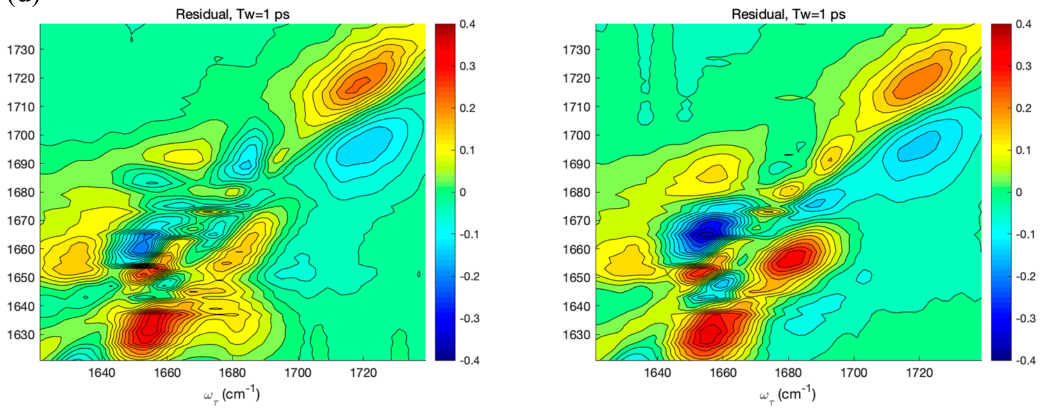

Residual, Tw=1 ps
Residual, Tw=1 ps
1730
1720
1710
1700
1690
1680
1670
1660
1650
1640
1630
1640
1660
1680
1700
1720
ω_τ (cm^-1)
0.4
0.3
0.2
0.1
0
-0.1
-0.2
-0.3
-0.4

(e)

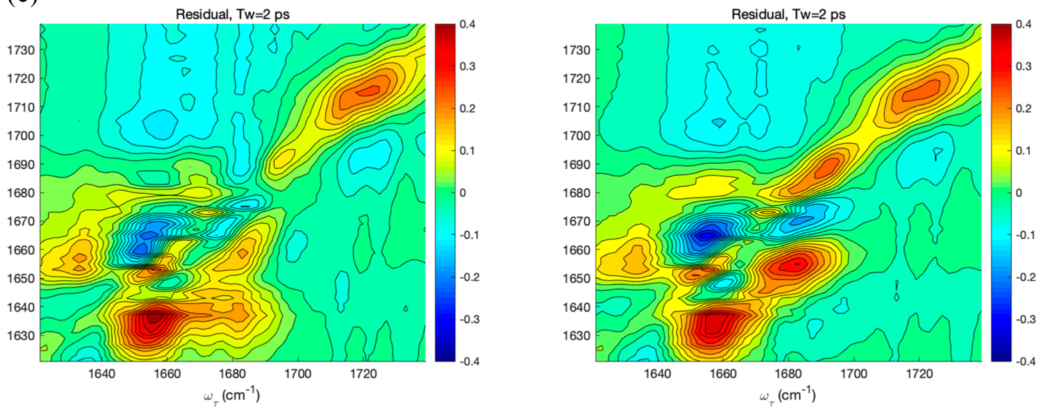

Residual, Tw=2 ps
Residual, Tw=2 ps
1730
1720
1710
1700
1690
1680
1670
1660
1650
1640
1630
1640
1660
1680
1700
1720
ω_τ (cm^-1)
0.4
0.3
0.2
0.1
0
-0.1
-0.2
-0.3
-0.4

(f)

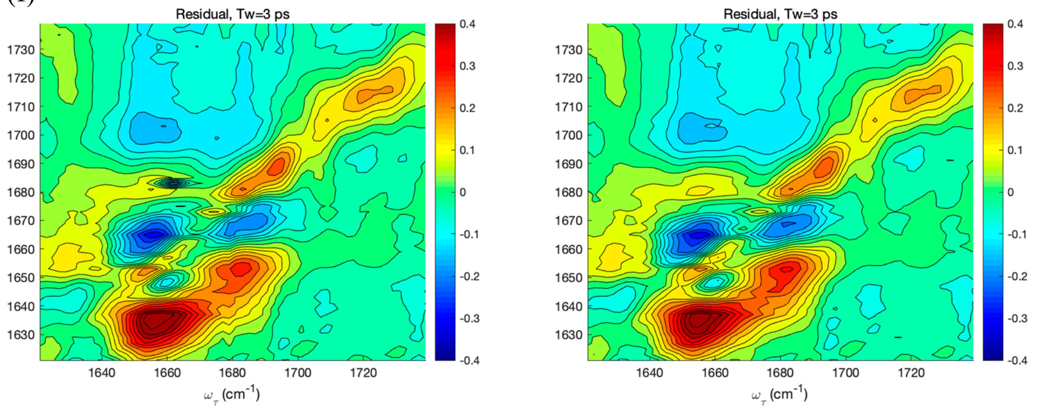

Residual, Tw=3 ps
Residual, Tw=3 ps
1730
1720
1710
1700
1690
1680
1670
1660
1650
1640
1630
1640
1660
1680
1700
1720
ω_τ (cm^-1)
0.4
0.3
0.2
0.1
0
-0.1
-0.2
-0.3
-0.4

(g)

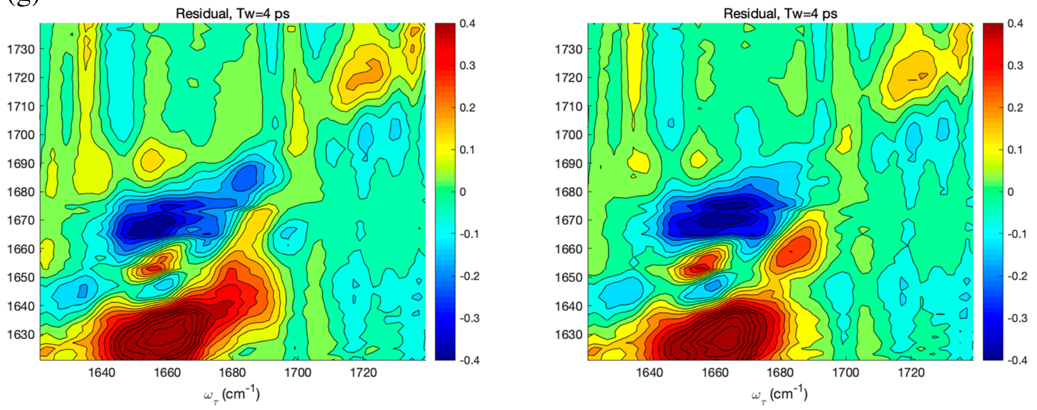

Residual, Tw=4 ps
Residual, Tw=4 ps
1730
1720
1710
1700
1690
1680
1670
1660
1650
1640
1630
1640
1660
1680
1700
1720
ω_τ (cm^-1)
0.4
0.3
0.2
0.1
0
-0.1
-0.2
-0.3
-0.4

(h)

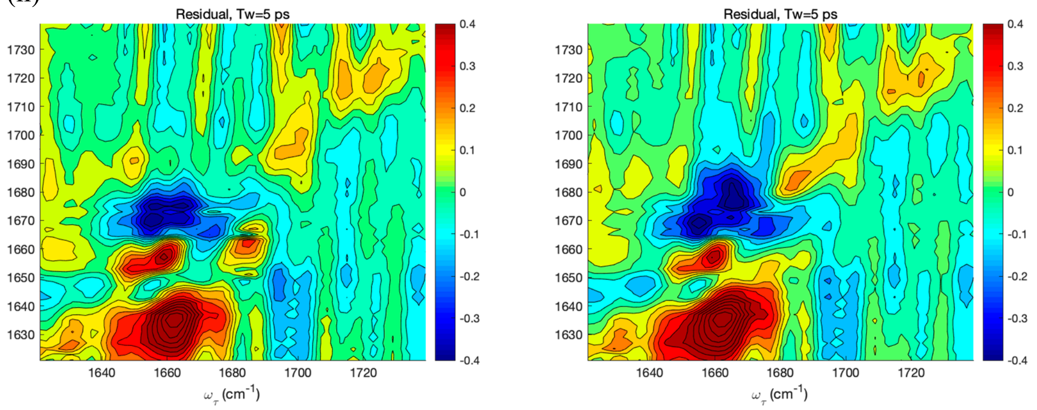

(i)

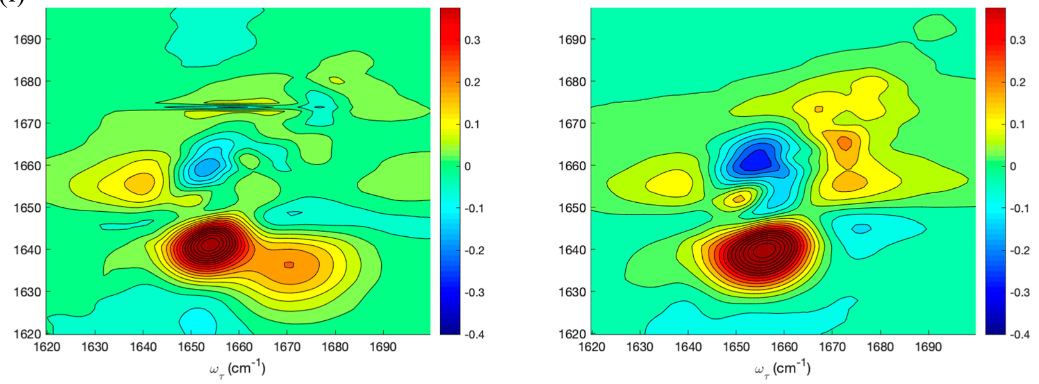

(j)

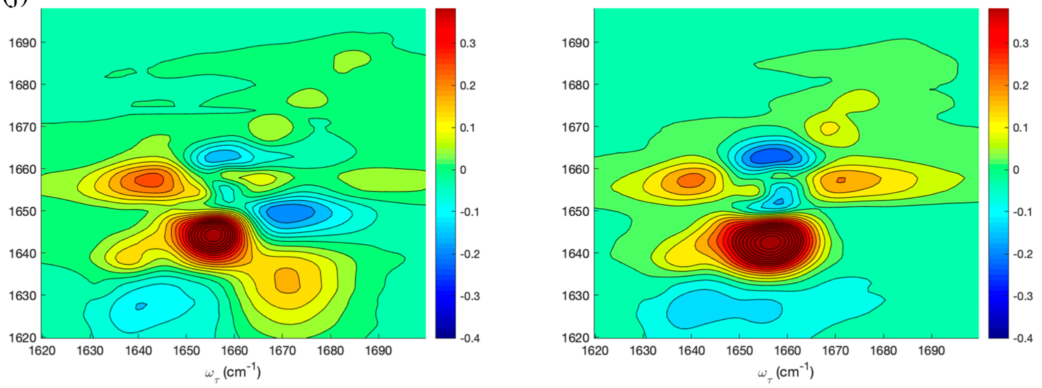

Figure S2. Residuals obtained from volume-fitting, defined as the original spectra minus the fit spectra, for wait times of (a) 0.2 ps, (b) 0.5 ps, (c) 0.7 ps, (d) 1 ps, (e) 2 ps, (f) 3 ps, (g) 4 ps, (h) 5 ps, and for computed spectra at wait times of 0 for (i) 1fmv and (j) 1vom. Residuals corresponding to fitting with cross-peaks are shown on the left, and those without cross peaks on the right.

Table S1. Fitting parameters for Gaussian fits of experimental 2D-IR spectra.

Cross-peaks	T_w(ps)	Amp	center_ω_τ	center_ω_m	width_ω_τ	width_ω_m	Anharmonicity
yes	0.2	13.7	1655	1656	10	20	16
		6.1	1682	1684	6	13	10
		5.8	1665	1685	13	13	10
		4.6	1660	1656	12	7	10
no	0.2	24.3	1657	1655	10	17	10
		9.4	1674	1685	13	13	10
yes	0.5	12.2	1655	1655	9	13	13
		7.0	1682	1680	8	13	11
		1.8	1656	1683	6	5	16
		3.3	1678	1661	10	13	14
no	0.5	11.4	1655	1655	9	13	13
		4.8	1679	1679	8	13	20
yes	0.7	8.8	1655	1655	8	13	14
		5.7	1683	1680	8	13	10
		1.2	1656	1682	6	6	13
		2.4	1678	1664	11	13	19
no	0.7	8.8	1656	1655	9	13	14
		3.7	1679	1679	9	13	20
yes	1	6.3	1655	1655	9	13	14
		3.8	1682	1679	8	13	11
		0.8	1656	1684	8	4	17
		1.7	1678	1662	10	13	17
no	1	6.2	1656	1655	10	13	14
		2.5	1680	1679	9	13	20
yes	2	2.4	1655	1655	9	12	13
		1.3	1683	1678	7	11	11
		0.2	1658	1684	7	3	20
		0.9	1678	1661	12	11	17
no	2	2.4	1656	1655	9	12	13
		1.1	1679	1671	10	13	16
yes	3	1.1	1656	1655	9	11	14
		0.4	1679	1670	11	13	20
		0.3	1662	1683	3	0	14

		0.0	1679	1655	0	13	17
no	3	1.0	1655	1655	9	11	14
		0.5	1678	1669	10	13	20
yes	4	0.5	1658	1658	12	16	20
		0.3	1685	1681	6	10	11
		0.0	1670	1685	8	5	18
		0.2	1683	1664	10	10	17
no	4	0.8	1662	1655	14	18	12
		0.2	1683	1681	7	12	16
yes	5	0.4	1661	1662	13	17	17
		0.1	1684	1683	4	7	19
		0.0	1655	1680	6	12	15
		0.2	1685	1665	3	0	14
no	5	0.7	1661	1659	13	18	10
		0.0	1660	1685	8	4	11

Table S2. Fitting parameters for Gaussian fits of computed 2D-IR spectra.

Model	Cross-peaks	T_w(ps)	Amp	center_ω_τ	center_ω_m	width_ω_τ	width_ω_m	Anharmonicity
1fmv	yes	0	16.2	1654	1653	5	6	8
			2.5	1675	1674	5	5	9
			1.7	1658	1674	10	0	9
			4.4	1670	1656	10	10	15
	no	0	18.1	1655	1652	7	8	8
			1.7	1673	1673	3	3	8
1vom	yes	0	16.9	1656	1655	5	5	8
			0.3	1674	1675	4	4	13
			5.5	1655	1674	9	0	11
			4.2	1670	1651	10	10	15
	no	0	17.6	1656	1654	6	6	8
			0.0	1679	1674	1	3	10
